# Enhancing behavioral sleep care with digital technology: study protocol for a hybrid type 3 implementation-effectiveness randomized trial

**DOI:** 10.1186/s13063-020-04974-z

**Published:** 2021-01-11

**Authors:** Anne Germain, Rachel R. Markwald, Erika King, Adam D. Bramoweth, Megan Wolfson, Gilbert Seda, Tony Han, Erin Miggantz, Brian O’Reilly, Lars Hungerford, Traci Sitzer, Vincent Mysliwiec, Joseph J. Hout, Meredith L. Wallace

**Affiliations:** 1 NOCTEM, LLC, 218 Oakland Avenue, Pittsburgh, PA 15213 USA; 2 Warfighter Performance Department, Naval Health Research Center, 140 Sylvester Rd, San Diego, CA 92106 USA; 3 Mental Health Division, Air Force Medical Readiness Agency, 2261 Hughes Ave, Suite 153, JBSA Lackland AFB, TX 78236-9853 USA; 4 VA Pittsburgh Healthcare System, Research Office Building (151RU), University Drive C, Pittsburgh, PA 15240 USA; 5 Naval Medical Center San Diego, 34800 Bob Wilson Dr, San Diego, CA 92134 USA; 6 Leidos, Inc., 4161 Campus Point Ct., San Diego, 92121 USA; 7 Madigan Army Medical Center, 9040A Jackson Ave, Joint Base Lewis-McChord, WA 98431 USA; 8 Defense and Veterans Brain Injury Center, Naval Medical Center San Diego, 34800 Bob Wilson Drive, San Diego, CA 92134 USA; 9 Division of Behavioral Medicine, Department of Psychiatry, UT Health San Antonio, 7703 Floyd Curl Drive, MC 7747, San Antonio, TX 78229-3900 USA; 10 Knowesis, Inc., 816 Camaron St. Suite 231, San Antonio, TX 78212 USA; 11 University of Pittsburgh, 3811 O’Hara Street, Pittsburgh, PA 15213 USA

**Keywords:** Digital health technologies, Insomnia, Behavioral sleep medicine, Military personnel, Veterans, Effectiveness, Implementation facilitation, Cognitive-behavioral therapy for insomnia

## Abstract

**Background:**

Insomnia affects almost one in four military service members and veterans. The first-line recommended treatment for insomnia is cognitive-behavioral therapy for insomnia (CBTI). CBTI is typically delivered in-person or online over one-to-four sessions (brief versions) or five-to-eight sessions (standard versions) by a licensed doctoral or masters-level clinician with extensive training in behavioral sleep medicine. Despite its effectiveness, CBTI has limited scalability. Three main factors inhibit access to and delivery of CBTI including restricted availability of clinical expertise; rigid, resource-intensive treatment formats; and limited capacities for just-in-time monitoring and treatment personalization. Digital technologies offer a unique opportunity to overcome these challenges by providing scalable, personalized, resource-sensitive, adaptive, and cost-effective approaches for evidence-based insomnia treatment.

**Methods:**

This is a hybrid type 3 implementation-effectiveness randomized trial using a scalable evidence-based digital health software platform, NOCTEM™’s Clinician-Operated Assistive Sleep Technology (COAST™). COAST includes a clinician portal and a patient app, and it utilizes algorithms that facilitate detection of sleep disordered patterns, support clinical decision-making, and personalize sleep interventions. The first aim is to compare three clinician- and system-centered implementation strategies on the reach, adoption, and sustainability of the COAST digital platform by offering (1) COAST only, (2) COAST plus external facilitation (EF: assistance and consultation to providers by NOCTEM’s sleep experts), or (3) COAST plus EF and internal facilitation (EF/IF: assistance/consultation to providers by NOCTEM’s sleep experts and local champions). The second aim is to quantify improvements in insomnia among patients who receive behavioral sleep care via the COAST platform. We hypothesize that reach, adoption, and sustainability and the magnitude of improvements in insomnia will be superior in the EF and EF/IF groups relative to the COAST-only group.

**Discussion:**

Digital health technologies and machine learning-assisted clinical decision support tools have substantial potential for scaling access to insomnia treatment. This can augment the scalability and cost-effectiveness of CBTI without compromising patient outcomes. Engaging providers, stakeholders, patients, and decision-makers is key in identifying strategies to support the deployment of digital health technologies that can promote quality care and result in clinically meaningful sleep improvements, positive systemic change, and enhanced readiness and health among service members.

**Trial registration:**

ClinicalTrials.gov NCT04366284. Registered on 28 April 2020.

## Background

This implementation-effectiveness hybrid type 3 trial [1, 2] aims to implement a large-scale, cluster-randomized pragmatic demonstration to inform behavioral sleep medicine practices and policies for active duty service members (ADSMs), veterans, and other beneficiaries with chronic insomnia disorder who receive care in military treatment facilities (MTFs) and affiliated clinics and wellness centers. Chronic insomnia disorder is defined as difficulty falling or staying asleep that persists for at least 3 months and is associated with daytime impairments [3]. These impairments span a variety of domains of functioning including memory and concentration difficulties, irritability, somatic symptoms, worry, and absenteeism. Insomnia is endemic among military personnel as well as veterans. Depending on the measurement method (e.g., self-report, sleep diary, diagnostic interview), insomnia affects between 24 and 90% of ADSMs and veterans who have deployed since 2001 [4–7]. Insomnia is also prevalent among non-deployed ADSMs [8–10].

Insomnia not only degrades military readiness, but also hinders resilience, quality of life, mood, and overall health [4]. In fact, insomnia is a robust risk factor for poor psychological and physical health outcomes [4–10], and is often comorbid with psychiatric and medical conditions such as posttraumatic stress disorder (PTSD), depression, suicidality, anxiety, alcohol use disorder, hypertension, chronic pain, obesity, and diabetes [5–14]. In the military population, insomnia symptoms have been associated with decreased resilience [15]. In a large study which evaluated 55,201 military personnel, those with insomnia symptoms reported lower self-rated health, were less likely to deploy, had increased days of missed work and healthcare utilization, and were more likely to be discharged early from the military [15].

The first-line recommended treatment for insomnia is cognitive-behavioral therapy for insomnia (CBTI) [16–19]. CBTI is typically delivered in-person (including via tele-health) over one-to-four sessions for brief versions, or five-to-eight sessions for the standard version, by a licensed doctoral or masters-level clinician with extensive training in behavioral sleep medicine [20–25]. Both brief and standard CBTI approaches yield comparable clinically meaningful improvements in insomnia severity [21, 26–30], with effect sizes in the moderate-to-very large range (Cohen’s *d* coefficients between .50 and 2.15). Response rates are also remarkable. For example, the Veterans Health Administration (VHA) national rollout of CBTI [14, 31] reported that, at post-treatment, 73% of 431 treated veterans endorsed Insomnia Severity Index [32] scores below the cutoff for moderately severe insomnia (ISI <  15), and that 35% no longer met criteria for insomnia (ISI < 8). A previous clinical trial with veterans undergoing a 4-week CBTI protocol reported response rates over 75% and remission rates of more than 50% [22]. CBTI has been shown to be effective for chronic insomnia, including insomnia that is comorbid with PTSD, anxiety, depression, alcohol and substance use disorders, suicidality, and chronic pain in both civilian and military samples [21, 33, 34]. Importantly, improvements in insomnia are maintained over time [35].

Despite its effectiveness, the limited scalability of CBTI impedes access to and delivery of evidence-based behavioral sleep medicine (EB-BSM) and contributes to the continuance of hypnotics as a common insomnia treatment. In a comprehensive study of sedative hypnotic medications in the military health system, ADSMs were significantly more likely than non-ADSMs to be prescribed this class of medications [30]. Notably, the ADSMs were markedly younger at 33.5 years of age compared to 59.1 years in the non-ADSMs. Approximately 10% of 18,000 surveyed ADSMs reported using sleep aids daily or almost daily. Their abuse potential, side effects, and link to increased risk of injurious behaviors raise serious health and safety concerns for all ADSMs due to their high-risk occupations [36–39].

Three main factors inhibit access to and delivery of CBTI to meet the needs of ADSMs. First, there is restricted availability of clinical expertise. Specialty sleep care clinics and expert behavioral sleep medicine providers are not readily available in the civilian sector [40–42] and across military treatment facilities (MTFs) and clinics, or in the more than 150 countries where US Armed Forces are stationed. Despite efforts to increase the number of providers trained in CBTI, shortfalls related to provider and geographic availability remain (e.g., many providers are clustered near urban, academic medical centers). Second, CBTI delivery formats (in-person or via telemedicine) are resource intensive. Even when available, the in-person delivery format of CBTI often creates barriers to receiving or adhering to treatment visit schedules due to travel distance (to and from the clinics), conflict with work and family schedules, childcare availability, and incompatibility with the demanding operational tempo of ADSMs [43]. To mitigate some of these barriers, online programs have been developed [44]. Online programs are self-administered (or unguided), and delivered without interaction with a dedicated clinician [43, 45–48]. Although these approaches are efficacious, they still require non-negligible time commitments from patients for several weeks to several months [43, 47–49], and attrition can be higher compared to clinician-assisted CBTI programs [43, 46, 49–51]. Third, there are limited capacities for just-in-time monitoring and treatment personalization. The current CBTI delivery formats (in-person or online) rely on scheduled weekly or bi-weekly appointments along with completion of homework, with no or limited capabilities for real-time (or near real-time) monitoring of adherence, intervention, and patient-clinician engagement. The available CBTI formats limit providers’ ability to prospectively monitor adherence, side effects, and improvements over time so that just-in-time recommendations can be delivered, and non-adherence or exacerbation of symptoms can be detected and addressed in a timely manner, which can enhance treatment outcomes. Furthermore, real-time patient-clinician interactions can augment patient engagement and the personalization and precision of care by allowing providers to adapt the frequency and duration of clinical engagement to best match individual patients’ needs (i.e., more complex patients may have more frequent or longer sessions compared to less complex patients). To address these challenges and effectively meet the insomnia needs of ADSMs, veterans, and beneficiaries, for evidence-based behavioral sleep medicine (EB-BSM) practices, innovative, scalable, personalized, resource-sensitive, adaptive, and cost-effective approaches are urgently needed.

Digital health (dHealth) technologies are becoming an increasingly popular means to overcome barriers related to the scalability, accessibility, and personalized delivery of evidence-based (EB) interventions. Some of the key current challenges in behavioral sleep medicine and advantages of leveraging dHealth technologies to overcome these challenges and support the delivery of EB-BSM are summarized in Table 1. Envisioned outcomes are also summarized in Table 1.

**Table 1:** Summary of challenges to dissemination of evidence-based behavioral sleep medicine and gap bridging solutions offered by digital technologies, and targeted outcomes

Current challenges	Solutions offered by dHealth technologies	Targeted outcomes
Access to evidence-based behavioral sleep care geographically limited to some urban/academic centers	Accessibility not limited by geographical locations	Leverage providers to serve more patients over broader catchment area
Limited number of trained behavioral sleep providers	Provide clinical decision-making support for non-sleep providers and consultation with experts in behavioral sleep medicine	Harness patient preference for treatment delivery format [40]
Delivery of care limited to in-person or synchronous telemedicine encounters	Delivery of synchronous and asynchronous care to match “just-in-time” patient needs and clinical resources	Increased accessibility for individuals with restricted mobility, and individuals with responsibilities that conflict with typical office hours (e.g., childcare, work schedule)
Cost-effectiveness	Independent of brick-and-mortar locations and related costs	Enhanced workflow and patient volume with minimal impact on workload
	Harness non-traditional workforce in behavioral health	
Fixed treatment protocols, appointment schedules, and communication means	Timely and secure just-in-time communication and exchange of information between provider and patient	Optimize personalization of assessment and intervention plan, prospective monitoring of adherence, side effects, progress, and outcomes

We first iteratively developed a prototype of the dHealth sleep platform with 5 providers and research clinicians and 17 military ADSM and veterans with chronic insomnia and comorbid psychiatric symptoms and conditions including PTSD, depression, and/or anxiety. Following this prototype, we evaluated the usability of the dHealth system by research clinicians and changes in sleep and daytime psychiatric symptoms in a sample of 27 ADSM and veterans. We compared these findings with results from previous trials where brief or traditional CBTI was delivered in person to a comparable sample of ADSM and veterans. This preliminary work strongly suggests that delivering EB behavioral insomnia treatments using dHealth technology is acceptable and feasible [52], associated with clinically meaningful improvement in insomnia severity (Cohen’s *d* effect sizes = 1.93), as well as improvements for the daytime PTSD symptoms (Cohen’s *d* effect sizes = 1.19), depression (Cohen’s *d* effect sizes = 1.13), and anxiety (Cohen’s *d* effect sizes = 0.74) [27]. Furthermore, these clinically significant improvements in insomnia and daytime function were the result of an average 15 min of clinical interaction, total, over 4 weeks between each participant and their research clinician. This contrasts sharply with the two-to-eight 30–45-min sessions typically required by in-person or tele-health CBTI (total time of 90 to 360 min). A potential reduction in clinical time required per patient of 88 to 96% suggests a substantial improvement in cost-effectiveness and scalability without compromising clinical outcomes.

A subsequent process improvement project in MTFs and affiliated clinics with 19 providers and over 100 patients support the acceptability and feasibility of deploying dHealth technologies such as NOCTEM’s Clinician-Operated Assistive Sleep Technology (COAST™) in real clinical settings (unpublished data) with behavioral health providers outside of sleep specialty care clinics. However, demonstration of effective deployment and implementation strategies as well as clinical outcomes associated with of COAST in military treatment facilities across the Department of Defense remains to be determined. This is the goal of this randomized hybrid type 3 implementation-effectiveness trial.

## Trial design

The hybrid type 3 implementation-effectiveness design [1, 2, 53] was selected because it primarily focuses on comparing different implementation methods. Randomization is at providers’ level, and the patient intervention is the same across the three facilitation conditions. This type of trial is uniquely suited to accelerate the transition of technology-supported EB-BSM practices tested in clinical research settings into real clinical and operational environments with ADSMs with chronic insomnia disorder and stress-related sleep disturbances. The proposed implementation-effectiveness hybrid design will yield insights into the most cost-effective strategies for implementing the COAST platform in MTFs, affiliated clinics, and wellness centers. The study can thus provide useful information to stakeholders (e.g., directors, administrators, other decision-makers) in evaluating the full potential of COAST to support access to and delivery of EB-BSM practices. Furthermore, this design maximizes the ecological validity and generalizability of findings.

**Fig. 1:**
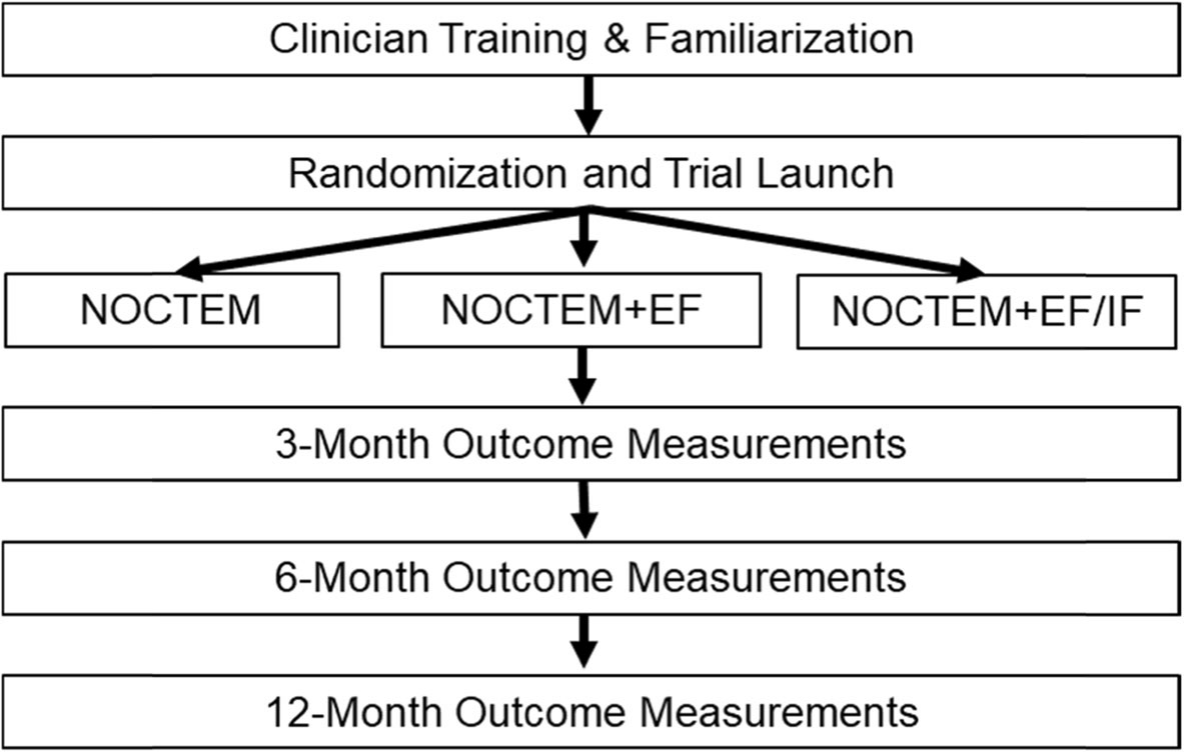
Design of the hybrid type 3 implementation-effectiveness trial

The investigative team has well-established relationships with key decision-makers and leaders, whereby the results of this trial can be directly translated into policy mandates and best practice guidelines across the Defense Health Agency (DHA). We employ an implementation science framework to evaluate the reach, adoption, and sustainability of digital sleep therapeutics in MTFs and affiliated clinics, which are expected to readily translate to broader veteran and civilian healthcare settings. The overall study design is summarized in Fig. 1.

## Trial status

This study received ethics review and approval from Solutions, IRB (Protocol #2019/12/10) on January 29, 2020; from the Naval Health Research Center (NHRC) on April 28, 2020 (Protocol #: NHRC.2019.0018); and from the US Army Medical Research and Development Command (USAMRDC), Office of Research Protections (ORP), Human Research Protection Office (HRPO) (HRPO Log Numbers E01442.1a [NOCTEM, LLC], E01442.1b [NHRC], E01442.1e [Naval Medical Center, San Diego], and E01442.1f [Naval Hospital Camp Pendleton]) on September 11, 2020.

## NOCTEM Clinician-Operated Assistive Sleep Technology (COAST™) platform

The NOCTEM COAST platform is designed to enhance the scalability of EB-BSM practices. Briefly, COAST is a software that encompasses clinician-centered dHealth clinical decision support algorithms and tools, a patient app, and a secure text messaging system. COAST is a cross-platform system that runs on any smart device (Android, iOS, Windows, etc.). Proprietary algorithms utilize self-report data collected daily from patients to identify disordered sleep patterns, which then guide personalized recommendations for intervention to the clinician. Sleep disturbances consistent with insomnia, nightmares, racing thoughts, daytime fatigue, sleepiness, and circadian disturbances can be detected, and evidence-based recommendations for behavioral sleep modifications are suggested to the clinicians, who then push the recommendations to the patient. The COAST tools were refined through iterative development and testing with end users, providers, and other key stakeholders in the DHA. COAST leverages patients’ preference for dHealth-based treatment approaches [54, 55], promotes engagement in treatment [56], and reduces the burdensome requirements of traditional CBTI by bringing the tools and clinical expertise directly to their smart device. These drivers also align directly with the DoD’s recognition of the unique capabilities of dHealth technologies in providing healthcare to ADSMs [57].

## Objective, aims, and hypotheses

The overarching objective of this investigation is to compare and evaluate three facilitation strategies for implementation (i.e., no facilitation, external facilitation only, and external plus internal facilitation) of the NOCTEM COAST™ platform in MTFs and clinics with a representative sample of ADSMs with common comorbid disorders. The specific aims are to (1) examine and compare the reach, adoption, sustainability, and magnitude of changes in a patient’s sleep for a new patient- and clinician-centered digital platform (COAST) alone, compared to COAST plus external facilitation (COAST+EF) or COAST plus EF and internal facilitation (COAST+EF/IF) in MTFs and affiliated clinics, and (2) quantify the magnitude of improvements in insomnia for patients who receive behavioral sleep care via the COAST platform.

Our first hypothesis is that providers randomized to the COAST+EF or COAST+EF/IF conditions will show greater (a) reach, (b) adoption, and (c) maintenance (sustainability) at 3- and 12-month follow-ups compared to providers randomized to the COAST-only condition. Our second hypothesis is that (a) COAST+EF and COAST+EF/IF will yield greater improvements in insomnia than COAST only over the acute 6-week intervention period, and (b) COAST+EF will not be inferior to COAST+EF/IF as measured by changes on the ISI over the acute 6-week intervention period.

To test these hypotheses, we will enroll at least 24 providers and 864 patients presenting with insomnia who are currently being seen at MTFs, affiliated clinics, and wellness centers across military branches. For an ecologically valid assessment of COAST’s potential to be implemented across facilities of the DHA, we will enroll providers who deliver services to ADSMs with comorbid PTSD, traumatic brain injury (TBI), chronic pain, and other psychiatric and medical conditions that are often comorbid with sleep disorders. The comparison of the three implementation strategies will provide insights into the advantages and disadvantages of each relative to patient- and clinician-centered outcomes. The results will inform anticipated larger implementation efforts across different clinical and operational settings within and outside the DHA.

## Selection criteria and enrollment of participating providers and patients

The trial aims to recruit at least 24 providers (clinicians and health coaches) who currently provide services in behavioral health to service members, their dependents, and beneficiaries across Navy Medical Forces Pacific (NMFP). The participating military treatment facilities (MTFs) and affiliated clinics are all located in Southern California. We aim to enroll providers who already deliver cognitive-behavioral health interventions as part of their regular practices, including behavioral sleep interventions like CBTI.

Each participating provider will enroll at least 36 patients over the course of 12 months, for a total minimum patient sample of 864. Across participating sites, providers will be informed of the trial through emails, newsletters, flyers, word of mouth, notifications sent to and by clinic leaders and/or department administrators, and flyers posted around the facilities and clinics. Providers will also be invited to participate in two remote half-day training workshops for which continuing education credits will be offered. The first segment of the training will be open to all interested providers at each site and focused on principles and practices of BSM.

Among providers who complete the first segment of the training, those who wish to participate in the trial will be invited to join the second segment of the training for a hands-on workshop on how to use the COAST platform. All interested providers will be eligible to enroll in the trial as long as they completed the first half of the workshop (or similar workshop in the past 6 months), evaluate and/or treat patients with insomnia, use cognitive-behavioral treatments as part of their practice, and provide electronic informed consent to participate in the trial. Providers from various backgrounds (e.g., clinical psychology, counseling, physician assistants, social work, physicians, nurses) will be eligible. We elected to enroll all eligible and interested credentialed providers, with at least a masters’ degree and training in the cognitive-behavioral therapeutic approach, and who are currently delivering care to military service members in general or specialized behavioral health clinics (e.g., concussion care clinics, pain management clinics, depression/anxiety treatment programs, sleep medicine clinic) to enroll a representative sample of providers across MTFs. Information regarding years of experience in behavioral health, experience in behavioral sleep medicine, professional degree, and clinical certification(s), as well as expectations about the use of technology in clinical settings will be collected at the onset of the study for all participating clinicians. While the study is not powered for definitive determination of the effects of each of these variables on implementation measures or patient outcomes, planned exploratory analyses will inform future implementation trials.

Prior to the COAST training workshop, all registered providers will receive an electronic copy of the consent to read and review before the training session. They will be encouraged to reach out to the study investigators or coordinators if they have questions. The consent form will be reviewed at the beginning of the COAST training workshop and questions will be addressed. Providers who decline to participate at that time will not be able to access the clinician portal nor use the COAST platform with their patients. Providers who elect to participate in the trial will provide consent by initializing each page and electronically signing the electronic consent form. A copy of the completed consent form is stored on the portal and can be downloaded by the provider. After obtaining informed consent to participate in the trial, information about providers’ background, current title, years of experience, familiarity with BSM and CBTI, and use of and proficiency with digital health tools will be collected.

During this workshop, and after, participating providers will receive access to the COAST clinician portal. During this segment of the workshop, providers will learn how to navigate both the COAST clinician portal and the patient NOCTEM app. After completing the training, providers will engage in a familiarization period with the clinician portal and app. Specifically, providers will be asked to use the COAST portal and app for a period of 10 to 14 days to become proficient with the COAST tools and workflow. During this trial period, the NOCTEM team will supervise providers’ use of the clinician portal and app. The NOCTEM team will offer consults, as needed, on the providers’ use of COAST and algorithm-driven recommendations, as well as address or communicate any technical issue that may arise during this time.

Once participating providers are familiar with the COAST platform, they will begin enrollment of eligible participants. Patients who are deemed eligible to use the NOCTEM app by their provider to support the management of insomnia and other behavioral sleep disturbances will be informed of the study. Clinicians who are investigators on the study or the assigned research coordinator will be able to enroll patients who express interest in using the NOCTEM app. Research coordinators and investigators who obtain consent from patients will follow a written script. The script follows the same structure as the electronic consent embedded in the NOCTEM COAST app. Following the script and electronic consent, the research staff member will review the study aims, procedures, risks, and alternatives prior to obtaining consent. Electronic consent will be obtained from patients via the app. For this, a waiver for documentation of consent has been granted. The research staff will then contact the treating clinician and inform him/her/them that their patient agreed to participate in the research study, is registered onto the clinician’s NOCTEM COAST portal, and is utilizing the app.

The sleep intervention will be delivered via the COAST digital sleep health platform to all participating patients. Exclusion criteria for participants are restricted to untreated sleep apnea [including less than 4 h of positive airway pressure (PAP) therapy per night]), currently pregnant or breast-feeding, parent of a newborn child, or a diagnosis of bipolar or psychotic disorders. The limited scope of exclusion criteria intends to optimize the generalizability of findings and representativeness of the sample relative to the larger population of ADSMs, veterans, and beneficiaries who receive care in MTFs and affiliated clinics of Navy Medical Forces Pacific. Participants who report clinically significant sleep disturbances despite concurrent pharmacological treatment with sedatives of hypnotics will be eligible for enrollment in the study if their provider deems it appropriate; discontinuation of medications is not required. There shall be no exclusion based on sex, minority status, or other health-related status (other than listed above). These exclusion criteria will be recommended to providers to avoid exacerbating excessive daytime sleepiness (for sleep apnea and/or pregnant women and new parents) and exacerbation of symptoms (for psychotic or bipolar disorders). Providers may also elect not to use the COAST platform, and refer a patient to primary care, psychiatry, sleep medicine, or another specialty care clinic for pharmacological sleep treatments if they deem it necessary for the patients. The ultimate decision regarding the inclusion of any patient will be left to their clinician, as s/he has the necessary clinical information to determine the best course of action for a given patient.

For patient enrollment, consent is obtained electronically via the NOCTEM app prior to activating the app. Electronic consent is an appropriate method for the present project because (1) COAST is a minimal risk care-enhancement project, (2) no protected health information (PHI) is stored on the app, and (3) no identifiable information will be collected via the portal or the app. After obtaining consent, patients will gain access to the patient app, including an orientation by the research team member provider on how to use the app and its assessment and messaging features. They will also be instructed to contact their provider via the secure text messaging system or by phone if they have any questions. No identifiable information will be visible on the patient app or clinician portal.

## Randomization of providers

After completing the COAST training and 10- to 14-day familiarization period, providers will be randomized in a 1:1:1 manner to one of three implementation strategies: COAST only, COAST+EF, or COAST+EF/IF (implementation strategies are further described below). Providers will be randomized within participating sites to one of the three facilitation conditions. A computer-generated randomization schedule based on a permuted block design will be created a priori. Using a permuted block design ensures that there will be equal numbers in each arm. The allocation will follow the generated sequence and the local research point of contact will inform the providers of their assignment at the end of the familiarization period, prior to patient enrollment.

## Intervention

Facilitation has been shown to enhance implementation of evidence-based treatments into practices [58, 59]. External facilitation (EF) refers to the addition of a key individual who is not embedded in the clinical setting, but who is tasked with supporting providers who are implementing new evidence-based practices, such as COAST in this trial. Because the external facilitator is not embedded in a single clinic, s/he may help in generating creative solutions for perceived and real barriers. Since an EF does not share clinical responsibilities and is not a supervisor or superior, s/he may also offer a supportive and guiding hand and be less hesitant to address difficult situations. This individual is available to troubleshoot as issues or questions arise and provide more interactive support, consultation, and feedback to providers. For the project, the NOCTEM team will provide EF via bi-weekly consult or supervision teleconference, or via telephone. The EF team will also be available by email and text messaging on an as-needed basis.

Internal facilitation (IF) refers to the presence of a champion embedded in the local system or clinic in which the evidence-based practice is being implemented, and who can serve as a more proximal facilitator in addition to the external facilitation. Compared to EF, IF has the advantage of providing a key resource who has a proximal relationship with providers and local administration, and who has in-depth knowledge of a clinic’s unique context, operational intricacies, clientele, etc. For the proposed trial, local sleep experts will provide IF; however, the IF will not be a study clinician. The comparison of the three implementation strategies will provide insights into the advantages and disadvantages of each of these three implementation interventions relative to patient-, clinician-, and system-level outcomes. The results will inform anticipated larger implementation efforts across different clinical and operational settings.

Prior to the enrollment of providers and patients, the NOCTEM team and the EFs and IFs will first complete an in-depth review of written guidelines and standard operating procedures for this project. During the trial, the EF and IF teams will meet monthly by phone and communicate via email, as needed, between scheduled monthly meetings to address any issues that arise and share newly acquired information. The EF and IF guidelines will be updated as needed to make sure both processes are documented and kept up to date during the trial.

The sleep intervention is the same for all patients enrolled by participating clinicians. When a disordered sleep pattern is detected by the NOCTEM algorithms (usually within 7 days), the provider will receive automated sleep recommendations for each patient and will be asked to review and confirm prior to forwarding it to the patient. Recommendations are consistent with stimulus control, sleep restriction, and other EB-BSM techniques, including suggested times for rise time and bedtime and exercise to minimize nightmares. Providers may elect to modify the algorithm-driven recommendations as they see fit, based on knowledge of their patients. Patients will receive their provider-approved, personalized behavioral sleep recommendations on a weekly basis. This process will be repeated for the next 4 to 6 weeks.

Participating patients will not be terminated from the study until (1) they decide to stop using the app; or (2) their provider deems that a different treatment or referral is required; or (3) the treatment course is complete, and the use of the app is no longer necessary; or (4) the end of the project. COAST is not commercially available, and patients who wish to remain in sleep treatment will be encouraged to discuss options with their provider. Because the COAST system requires that a provider monitors entries and sleep status and progress, the app will be disabled when a patient is no longer in the care of a provider. This will avoid misleading patients that they are still under the care of the provider through the app only.

Over the duration of the intervention, patients will be asked to complete daily sleep logs, measures of overall sleep quality, daytime psychiatric symptoms, perceived improvements, and side effects through the app. Symptoms of depression, anxiety, and PTSD will be assessed weekly with the PHQ-2 [60], GAD-2 [61], and PC-PTSD-5 [62], respectively. Side effects will be evaluated weekly using adapted version of the Asberg Side Effect Scale [63], whereas sleepiness will be assessed with the Epworth Sleepiness Scale [64, 65]. The main expected side effect is a transient increase in sleepiness. Side effect ratings from the modified Asberg Side Effect Scale and Epworth Sleepiness Scale are assessed weekly and available to providers for review to inform the titration of behavioral recommendations as needed. Self-perceived improvements will be assessed with the Patient Report Global Impression Scale [66]. Providers will be asked to complete the Clinician Global Impression Scale [66] on a weekly basis after reviewing patient-reported side effects and other symptom measures, and sleep diaries.

All data gathered in this project from providers and patients will be completed electronically, and data will be saved automatically on secure the NOCTEM cloud. Data completeness is facilitated at the time of completion by participating patients or providers by not allowing participating patient to skip questions (yet, one can decline to answer a question), and by reminders to complete incomplete forms sent via the app or portal. Automated data review and report will be generated to assess completeness and upload into the main database.

Data management, monitoring of the integrity of the interventions (i.e., facilitation conditions), training of facilitators, and local research assistants will be coordinated by the NOCTEM team. A Collaborative Research and Development Agreement (CRADA) is in place with site principal investigators so that data collected can be shared.

The NOCTEM cloud-based databases are protected by several procedures, including password protection of subject data and a firewall around the NOCTEM network. For the NOCTEM COAST platform, we have developed comprehensive privacy and security measures including unique activation personal identification numbers and passwords, secure socket layer (HTTPS/SSL) protocol, strong encryption, and compliance with the Defense Information Systems Agency (DISA) Security Technical Implementation Guides (STIG). All data are stored in a secure, HIPAA-compliant cloud server. Clinicians and participating patients will have unique research ID numbers that will be used in lieu of personally identifiable information for data collection and storage. The research ID numbers are unrelated to any potentially identifiable numerical series, such as social security number, medical record number, or date of birth. Study data will be kept strictly confidential, and participants’ identities will not be revealed in any publication.

## Outcome measures

Table 2 summarizes the schedule of enrollment, interventions, and assessments for participating providers. Clinician-centered, implementation outcomes from the reach, effectiveness, adoption, implementation, and maintenance (RE-AIM) framework [67], see also re-aim.gov, include reach, adoption, and maintenance (sustainability). Provider reach is defined as the ratio of providers who provide informed consent to participate and complete the COAST training over the number of providers who received information about the trainings via emails, personal invitations, and other communication means targeting behavioral health providers. Patient reach is defined as the ratio of patients who consent to use the app relative to the number of patients deemed eligible to use the app. Adoption is defined as the ratio of newly trained providers who initiate the use of the COAST platform with patients (i.e., provide a patient with an app code) over the total number of providers who completed the COAST training (i.e., trained users + trained non-users). Sustainability is determined by comparing the frequency of COAST platform use by the trained providers across the three groups at the 3-, 6-, and 12-month assessments to measure potential changes on these domains over time.

**Table 2:** Providers’ schedule of enrollment, interventions, and assessments for this hybrid type 3 implementation-effectiveness randomized trial

			Study period				
	Enrollment	Consent	Baseline	Post-randomization			
**Timepoint**	***-t***_***1***_	**0**	***t1***	***Familiarization***	***3 months***	***6 months***	***12 months***
**Enrollment:**	X						
Complete sleep training	X						
Informed consent		X					
NOCTEM COAST training			X				
Randomization			X				
**Interventions:**							
COAST only							
COAST + external facilitation (EF)							
COAST + EF + internal facilitation (EF/IF)							
**Assessments:**							
Demographics and practice survey			X				
Post-training survey			X				
Organization readiness for implementing changes			X			X	X
System usability scale			X	X		X	X

Fidelity is evaluated by quantifying the adherence (or discrepancy) between algorithm-recommended interventions relative to interventions that are sent to patients by providers. As in some cases there may be valid clinical reasons why a provider determines a patient should not receive the algorithm-recommended intervention (e.g., sickness, incompatible with assigned duties), we expect a rate of non-adherence of approximately 20%—noting we will not be accessing patients medical records to validate the potential reasons for deviations.

The primary patient-centered outcome measure of treatment effectiveness is the Insomnia Severity Index (ISI [32]). Other symptom domains to be assessed are summarized in Table 3, and include the PTSD Checklist-5 (PCL-5 [47, 48]), the 8-item Patient Health Questionnaire (PHQ-8 [49]) to assess symptoms of depression, the 7-item Generalized Anxiety Disorder scale of the PHQ (GAD-7 [50]), the PEG, a 3-item validated and reliable pain measure [68], and the 4-item PROMIS Satisfaction with Social Roles and Activities [69]. For patients, the total time required to complete the battery is approximately 20 min. The battery will be completed through the NOCTEM app at baseline, post-treatment, and again 3 and 12 months post-treatment.

**Table 3:** Self-report measures of sleep and daytime symptoms to be completed by participating patients at baseline, post-intervention, and at the follow-up assessments

Domain	Scale	Number of items	Time to complete
**Sleep**			
Insomnia	Insomnia Severity Index (ISI)	7	< 3 min
Perceived global improvement	Patient self-report Global Improvement Scale (PGI)	1	< 1 min
Clinician-rated global improvement	Clinician-rated Global Improvement Scale (CGI)	3	< 3 min
**Daytime symptoms**			
Daytime sleepiness	Epworth Sleepiness Scale (ESS)	8	< 1 min
Posttraumatic stress disorder (PTSD)	PTSD Checklist (PCL-5)	20	< 3 min
Depression	Patient Health Questionnaire (PHQ-8)	8	< 3 min
Anxiety	Generalized Anxiety Disorder (GAD-7)	7	< 3 min
Pain	Pain, Enjoyment, General activity (PEG)	3	< 1 min
Functioning	Satisfaction with Social Roles and Activities	4	< 1 min

## Statistical analyses plan and power calculations

Preliminary data analyses (e.g., descriptive statistics, correlations, principal component analysis) will be performed prior to hypothesis testing to check for collinearity of covariates and outcome measures, to ensure completeness and accuracy of data, and to evaluate distributions. Missing observations will be investigated to determine the underlying mechanism (missing at random, completely at random, not at random) so that appropriate methods for handling the missing data can be employed. Namely, if data are missing at random, we will use multiple imputation (MI) to ensure that any individuals with missing covariates or outcome data can be included in the analysis sample. However, if data are found to be missing not at random, we will use methods that explicitly model this type of nonrandom missingness (e.g., pattern mixture models) and perform sensitivity analyses evaluating the impact of the missingness. Power was computed using PASS statistical software and documentation. Each model will include covariates including study site, patient age and sex, military rank, and use of psychotropic medication (yes/no). Masked data will be provided to the biostatistician who will perform the analyses. Randomization will be unmasked only after analyses are finalized.

The first hypothesis is that providers randomized to one of the two COAST+ conditions will show greater reach, adoption, and sustainability at the 3- and 12-month follow-ups compared to providers randomized to the COAST only. For adoption, we will use a test for two proportions to determine whether reach and adoption differ between COAST versus COAST+EF and COAST+EF/IF at the 3- and 12-month follow-ups. With at least 8 providers in COAST and 16 in COAST+EF and COAST+EF/IF, as well as a reach and adoption between 0.50 and 0.60 in COAST, we will have 0.80 power (alpha = 0.05) to detect a difference in proportions between COAST and COAST+ of 0.39–0.46. For sustainability, each provider’s average number of interactions per patient over time and number of newly enrolled patients each month for each of the 12 months will be computed. We will then fit a generalized linear mixed effects model to regress number of interactions on group (COAST versus COAST+) and time. The group-by-time interaction will also be tested. Models will include a random intercept and time effect. With six repeated measures (autocorrelation 0.60–0.80) and 24 providers (8 COAST and 16 COAST+EF and COAST+EF/IF), we expect 0.80 power (alpha = 0.05) to detect differences of 0.99–1.11 between groups. With 12 repeated measures, we expect power to detect differences of 0.97–01.10 between groups.

The second hypothesis is that COAST+EF and COAST+EF/IF will yield greater improvements in patient insomnia symptom severity than COAST only over the acute 6-week intervention period. We will compute the change in ISI from baseline to 6 weeks (∆ISI = baseline ISI − 6-week ISI), with higher ∆ISI values indicating a greater improvement over time. We will use a mixed effects model to regress ∆ISI on group, accounting for nesting within providers through a random effect and including relevant covariates. We will extract estimates from the model to compute the standardized mean difference *d* [70, 71] and 95% confidence interval between COAST and COAST+ (EF; EF/IF) accounting for patients nested within providers. Second, using a mixed effects model with a random clinician effect, we will regress ISI at baseline and 6 weeks of treatment (COAST versus COAST+EF and COAST+EF/IF), time, and their interaction, controlling for relevant covariates. A significant treatment-by-time effect will indicate that the change in ISI differs between COAST and COAST+EF and COAST+EF/IF. Assuming an average of 12 providers per group (8 in COAST and 16 in COAST+EF and COAST+EF/IF) and 36 patients per clinician, we expect 0.80 power (alpha = 0.05) to detect a standardized mean difference of 0.232 between groups. We will also evaluate the extent to which COAST+EF is not inferior to COAST+EF/IF as measured by changes on the ISI over the acute 6-week intervention period. This non-inferiority hypothesis will be tested with the general form—H_0_: COAST+EF is inferior to COAST+EF/IF versus H_1_: COAST+EF is non-inferior to COAST+EF/IF. Using the methods described above, we will compute the standardized mean difference *d* and 95% confidence interval [*d* (95% CI)] for COAST+EF − COAST+EF/IF of ∆ISI. As positive values of ∆ISI indicate more improvement, a positive *d* is in favor of COAST+EF and a negative *d* is in favor of COAST+EF/IF. We will use a non-inferiority margin (NIM) of − 0.50 and declare COAST+EF to be non-inferior to COAST+EF/IF if the lower bound of the 95% CI for *d* is > − 0.50. We will declare COAST+EF to be inferior to COAST+EF/IF if the lower bound of the 95% CI for *d* is ≤ − 0.50. For example, if *d* (95% CI) = − 0.10 (− 0.40, 0.20), we would declare COAST+EF as non-inferior to COAST+EF/IF because − 0.40 > − 0.50. However, if *d* (95%CI) = − 0.40 (− 0.70, − 0.10), we will declare COAST+EF to be inferior because − 0.70 ≤ − 0.50. These one-sided decision rules equate to requiring the effect size of the COAST+EF to COAST+EF/IF comparison to have no more than moderate strength (|d| = 0. 5), in favor of COAST+EF/IF. The NIM of |d| = 0.5 set to determine non-inferiority is stringent as it represents 50% of the effect size reported in recent meta-analyses [50, 72, 73]. Assuming 8 providers per group and 36 patients per clinician, an intra-class correlation of 0.01 [74], a non-inferiority margin of 0.50, and a true difference of δ = 0, we expect > 0.99 power to reject our null hypothesis and conclude that COAST+EF is non-inferior to COAST+IF/EF.

## Secondary and exploratory analyses

Secondary analyses will explore the durability of symptom improvements over time among patients who used the COAST platform with their provider over time. While the time elapsed between the post-treatment at 6 weeks and discharge may vary across patients, we will, nevertheless, explore the extent to which detectable sleep improvements after 6 weeks may persist over time. The variability in elapsed time between the post-treatment and discharge assessment will be taken into consideration in the statistical analyses. We will first summarize and compare the differences in discharge times by examining relevant baseline characteristics (e.g., symptom severity, sleepiness, daytime impairments). We will then compute the average rate of change in ISI score per month [(6-week ISI − discharge ISI)/(discharge week − 6)]. Using a mixed model, we will fit an intercept-only model for the rate of change in ISI score, accounting for nesting within clinician through a random effect. An intercept that differs significantly from zero will indicate further change in ISI score between 6 weeks and discharge. Models will also include age and sex and other baseline characteristics that may be found to impact time to discharge. Finally, we will explore whether change in psychological distress (e.g., PHQ-8, GAD-7, PCL-5) corresponds to use of COAST by computing correlations between change in ISI score, and number of interactions with their assigned coach.

While the study is not powered for definitive determination of the effects of each of the clinician characteristics on implementation measures or patient outcome data, we will complete exploratory analyses to evaluate if these variables may impact outcomes of interest to inform future implementation efforts.

## Plan for dissemination of the results

We anticipate publishing the findings of the trial in at least one peer-reviewed publication and presenting primary and secondary data analyses at national scientific conferences, including the SLEEP meeting, the Military Healthcare System Research Symposium, and the American Telemedicine Association. Briefs delivered to Navy Medical leadership will also offer a path for dissemination of the study findings. Results of the trial will also be discussed with stakeholders and leadership in the DHA.

## Discussion

This implementation-effectiveness hybrid type 3 trial is innovative in its combination of digital technologies and evidence-based CBTI practices using a pragmatic approach and a primary focus on ecological clinical validity. This trial design was selected because it is uniquely suited to identify and guide future implementation and dissemination efforts to increase uptake of EB-BSM and improve sustainable access to care and to assess patient-centered outcomes. If successful, the utilization of a dHealth platform to support CBTI can have significant benefits such as improving the overall health, readiness, and resilience of the US Armed Forces; decreasing reliance on hypnotics; improving military retention rates; and decreasing insomnia-related healthcare costs. Scalable and cost-effective digital sleep solutions can have substantial benefits for the Veterans Health Administration as well as civilian healthcare providers and payors.

Based on our preliminary findings and other large-scale research and clinical efforts in the VA and [75, 76], we expect that reach, adoption, and sustainability (maintenance) will be enhanced through EF and EF/IF. We also expect that the magnitude of improvements in insomnia achieved through delivery of CBTI via the dHealth platform for patients enrolled in the trial will meet stringent criteria for clinical effectiveness. Overall, we expect that COAST implemented in MTFs, affiliated clinics, and wellness centers will be an acceptable and preferred care delivery model for delivering behavioral sleep interventions to ADSMs with insomnia and other behavioral sleep disorders.

There are, of course, some challenges that are anticipated over the course of the study. Changes in clinic leadership may impact the retention of enrolled clinics over the course of the trial. To mitigate this risk, we will work closely with each clinic’s leadership and stakeholders to solidify and maintain engagement. Similarly, the retention of providers may be challenging, as attrition naturally occurs due to relocations, promotions, leaves, or other factors. If a clinician withdraws from the trial, we will recruit, train, and replace them with a new provider to maintain a minimum of 24 enrolled providers. A prior implementation pilot revealed high variability among providers in adopting technology. To optimize proficiency and confidence with digital technology, we will closely supervise newly trained providers during the 10- to 14-day familiarization period to provide timely, personalized feedback, and to promote engagement.

While this trial will be conducted in MTFs and affiliated clinics and wellness centers, there is high translation potential to VA Medical Centers and Community-Based Outpatient Clinics as well as to civilian healthcare settings. In civilians, insomnia affects as many as 30% of the general population [3, 77]. In both veterans and civilians, insomnia is also associated with poor mental and physical health outcomes, absenteeism, heightened risk of accidents, decreased productivity, and increased healthcare costs and utilization [15, 78–84]. The same challenges of CBTI scalability, access, and delivery are found in VA and civilian healthcare sectors, and both have called for greater use of dHealth technologies to support high-quality care and reduce rapidly growing healthcare costs.

## Data Availability

Data sharing not applicable to this article as no datasets were generated or analyzed during the current study.
